# Detection of IgM Antibrucella Antibody in the Absence of IgGs: A Challenge for the Clinical Interpretation of Brucella Serology

**DOI:** 10.1371/journal.pntd.0003390

**Published:** 2014-12-04

**Authors:** Julián Solís García del Pozo, Santiago Lorente Ortuño, Elena Navarro, Javier Solera

**Affiliations:** 1 Department of Internal Medicine, Villarrobledo General Hospital, Villarrobledo, Spain; 2 Department of Microbiology, University General Hospital of Albacete, Albacete, Spain; 3 Research Unit, University General Hospital of Albacete, Albacete, Spain; 4 Department of Internal Medicine, University General Hospital of Albacete, Albacete, Spain; University of California, San Diego, School of Medicine, United States of America

## Abstract

The use of enzyme-linked immunosorbent assay (ELISA) for the detection of IgG and IgM antibodies antibrucella has become widespread in the diagnosis of human brucellosis. IgM anti-Brucella antibodies are indicative of acute infection. Between 2009–2013, 5307 patients were evaluated for serologic diagnosis at the Microbiology Laboratory of the Albacete General Hospital. A ELISA IgM-positive, IgG-negative anti-Brucella antibody serology pattern was detected in 17 of those patients. Epidemiology data, symptoms, laboratory data, treatment and outcome from these patients were reviewed. Sixteen patients presented with musculoskeletal pain, fatigue and/or fever and 1 was asymptomatic. Five patients received treatment with doxycycline combined with rifampin, gentamycin or streptomycin during 6–12 weeks, with no improvement. None of the 17 patients were finally diagnosed with brucellosis. Our results indicate that anti-Brucella IgM positive serology, per se, is not enough to diagnose acute brucellosis and other methods should be used for confirmation. Brucella serology data should be interpreted taking into account the patient's clinical history and epidemiological context.

## Author Summary

Human brucellosis may present with a wide variety of symptoms and signs. Adequate laboratory diagnostic methods become necessary. The diagnostic method that proves infection caused by *Brucella* spp. is the isolation of the bacteria from body fluids or tissues, but the difficulty in obtaining positive bacterial cultures has led to the development of other diagnostic techniques. Rose Bengal test, Wright agglutination, Coombs antibrucella, immunocapture techniques, and serology to detect specific IgG and IgM antibodies usually by enzyme-linked immunosorbent assay (ELISA), are the serological methods most often used for the diagnosis of brucellosis. The use of ELISA techniques has become widespread in recent years. The detection of specific IgM by using ELISA is considered indicative of acute or recent infection. However, IgM antibody detection in the absence of IgG may lead to an erroneous diagnosis of acute brucellosis. We described a series of patients in which serology detected IgM but not IgG antibrucella antibodies. In these patients the clinical picture and the outcome are not suggestive of active infection caused by *Brucella* spp. This finding supports the conclusion that *Brucella* serology data should be interpreted taking into account the patient's clinical history and epidemiological context.

## Introduction

Human brucellosis is a zoonosis with a worldwide distribution, with a low incidence in developed countries, but great importance in developing countries. Despite the efforts made to achieve its control or eradication, brucellosis remains prevalent in many countries of the Mediterranean area, the Middle East, lndia, Central Asia and Central and South America [1]. The disease may present with a wide variety of symptoms and signs. They include fever, chills, arthralgia, fatigue or lumbar pain. This broad spectrum of nonspecific symptoms makes diagnosis more difficult. Similar symptoms may be caused by other infectious diseases such as Q fever, *Salmonella* infections, tuberculosis or viral infections, and even non-infectious diseases [2]. For this reason adequate laboratory diagnostic methods to confirm the clinical suspicion become necessary.

The diagnostic method that proves infection caused by *Brucella* spp. is the isolation of the bacteria from body fluids or tissues. Although the isolation of *Brucella* spp. confirms the infection, the delay in culture results, the risk of infection of laboratory personnel [3] as well as the difficulty in obtaining positive cultures, has led to the development of other diagnostic techniques useful for the diagnosis of brucellosis. [4]. Standard agglutination test have the advantage of low cost, simplicity and general reproducibility. These characteristics have made it the reference serological method [5], [6]. In recent years, methods have been developed to detect the genetic material of the microorganism using polymerase chain reaction (PCR) techniques [7]. Even so, serological methods are most often used for the diagnosis of brucellosis. Among these serological methods are the Rose Bengal test, the Wright agglutination, the Coombs antibrucella, immunocapture techniques, and serology to detect specific IgG and IgM antibodies usually by an enzyme-linked immunosorbent assay method (ELISA) [8], [9].

ELISA techniques are low cost, require less time to complete and less training for interpretation compared with agglutination techniques [10]. These advantages explain their widespread use in recent years. However the sensitivity and specificity of ELISA for detection of antibodies against *Brucella* spp. differ among studies. Gomez *et al.* assign a sensitivity of 60% for IgM and 84% for IgG, while the combined specificity for IgG and IgM was 100% [11]. However Mantur *et al.* found a combined IgG and IgM ELISA sensitivity of 100% but a combined specificity of 71.3% [12]. Welch *et al.* reported a 92.3% combined sensitivity and a combined specificity of 55% [10]. The results of specific detection of IgG and IgM antibodies should be interpreted with caution [10], [11] since the antibody positivity is not always indicative of acute brucellosis, and its negativity does not exclude the disease.

The presence of specific IgM is considered indicative of acute or recent infection. However, IgM antibody detection in the absence of IgG may lead to an erroneous diagnosis of acute brucellosis [13] and may be a source of controversy. IgM antibodies can be detected because of cross-reaction in other clinical conditions, and also in the presence of rheumatoid factor. Pre-absorption of rheumatoid factor is required before the determination of IgM antibodies. [14].

Since April 2009, detection of IgG and IgM by ELISA has been introduced as a technique for serological diagnosis of human brucellosis at the Microbiology Laboratory of the Albacete General Hospital. This technique has replaced the Wright agglutinations and anti-Brucella Coombs test. Since then, some cases have been detected in which positive IgM results in the absence of antibrucella IgG have complicated the correct interpretation of the patient's symptoms, with the risk of misdiagnosis. The aim of this study is to assess the validity of the detection of IgM antibrucella when IgG is negative, by describing a series of patients in whom this serological pattern was found. We analyzed symptoms, medical history and clinical evolution, in order to determine whether these patients had acute brucellosis or not, and to better interpret such a result in clinical practice.

## Materials and Methods

We performed a search in the microbiology laboratory of the Albacete General Hospital for serum samples obtained between 2009 and February 2013 in which ELISA detected IgM antibrucella antibodies in the absence of IgG antibodies. This laboratory serves the Albacete General Hospital as well as the hospitals of Almansa, Villarrobledo and Hellin, all of them located in the Albacete Healthcare Region (Spain). The Albacete Healthcare Region has traditionally been an endemic area for brucellosis, although in the last 10 years the incidence has decreased significantly [15].

The medical records of these patients were thoroughly reviewed. Age, gender, history of epidemiological exposure to *Brucella* spp., symptoms that led to request serology, the number of times serology was repeated and the number of times in which the result was the same (IgG negative, IgM positive), the performance of other tests to confirm or discard the diagnosis of brucellosis, antibiotic treatment used following the results of the tests, and patient outcomes were collected. The final clinical diagnosis reached for each of these patients was recorded. Data were analyzed anonymously. The review of medical records was performed retrospectively on patient data and serological tests that were performed as part of routine hospital work. The Research Commission of the University Hospital of Albacete approved this study.

ELISA detection of IgG and IgM was performed using a commercial kit (Virion/Serion, Würzburg, Germany). The technique was performed according to the instructions from the manufacturer. In brief, 100 µl of diluted serum samples and ready to use control sera were added to the microtest wells with antigen. The samples were then incubated at 37°C for 60 minutes, after which the first wash was performed. Later, anti-human IgM or IgG conjugated with an enzyme (alkaline phosphatase) was added and incubated for 30 minutes at 37°C in a moist chamber. All wells were then washed to remove excess conjugate, followed by a new incubation for 30 min at 37°C with the enzyme substrate (paranitrophenylphosphate). Finally, the reaction was stopped by adding 100 µl of stopping solution. The enzyme reaction with the substrate yields a colored product. Color intensity is proportional to the amount of specific antibody and can be measured by photometric methods. In the case of IgM-detection, absorption of rheumatoid factor was performed following the manufacturer's instructions. Titers above 25 IU/ml were considered positive for IgG, and above 20 IU/ml were considered positive for IgM. Titers were considered uncertain between 25 and 20 IU/ml for IgG, and between 20 and 15 IU/ml for IgM. Sensitivity and specificity provided by the manufacturer were >99% and 99.3% respectively for IgG and 91.3% and >99% respectively for IgM [16].

## Results

From April 2009 (when the ELISA was introduced in our laboratory) to February 2013, ELISA serology was performed on 5307 patients, with a total of 6175 samples processed. Of these samples, 5703 samples were negative for both IgG and IgM, 10 samples were positive for both IgG and IgM, 394 samples were positive for IgG and negative for IgM, and 68 samples were negative for IgG and positive or uncertain for IgM detection. These latter 68 samples were from 26 patients. One of these patients had acute brucellosis with one determination that was positive for IgM and negative for IgG, but subsequent determinations were positive for both IgM and IgG. For the remaining 67 samples, 49 were positive for IgM detection and 18 were uncertain. These uncertain samples were not included in our study.

The 49 positive IgM samples were from 17 patients. The number of determinations per patient in whom serology was positive IgM and negative IgG ranged between 1 and 11. The age of these patients at the time of the first ELISA testing varied between 28 and 82 years. 35.3% of these patients were male. We have divided these patients into two groups. The first group included patients who had had brucellosis previously (Table 1), and the second group included patients who had never suffered from brucellosis (Table 2).

**Table 1:** Patients with previous brucellosis who had positive IgM and negative IgG antibrucella antibodies.

Patient	Age	Gender	Risk factors	Symptoms	Number of samples IgM + and IgG -	Other diagnostic tests	Antimicrobial treatment	Final diagnosis
1	54	F	Veterinarian (no recent contact with animals) Previous brucellosis	Asymptomatic (occupational medical check-up)	6	------------	No	none
2	82	F	Previous brucellosis (at age 20)	Low back pain	2	-------------	no	Spondylarthrosis, Left Renal Carcinoma
3	31	F	Previous brucellosis	Arthralgia, fatigue	11	Brucellacapt (positive), PCR (negative- 5 samples).	No	Treated brucellosis- probable chronic fatigue syndrome
4	56	F	Previous brucellosis	Arthralgia	1	PCR (negative 4 samples), serum agglutination (negative) Blood cultures (negatives)	no	Polyarthrosis. Treated Brucellosis
5	39	M	Previous brucellosis	arthralgia, fatigue	10	-------------	Dx (6 weeks) + STP (2 weeks)	Arthralgia. Treated Brucellosis.

Abbreviations: F: female; M: male; PCR: polymerase chain reaction; Dx. Doxycycline; STP: Streptomycin.

**Table 2:** Patients with no history of previous brucellosis who had positive IgM and negative IgG.

Patient	Age	Gender	Risk factors	Symptoms	Number of samples IgM + and IgG -	Rose Bengal test	Other diagnostic tests	Antimicrobial treatment	Final diagnosis
6	35	M	no	Fever and arthralgia	1	Negative	----------	no	Probable acute parvovirus infection
7	28	M	no	Oligoarthritis	7	Negative	PCR (negative)	Dx (6 weeks) + Stp (15 days)	Chronic oligoarthritis
8	50	V	no	Fever	1	Negative	-----------	Dx 100 mg/12 h (8 days)	Q fever
9	67	M	no	Pancytopenia and splenomegaly	1	Negative	-----------	No	lymphoproliferative disorder
10	46	M	no	Neck pain	1	Negative	-----------	No	Mechanical neck pain
11	61	M	no	Stroke (TIA). Right metatarsotarsiana Arthritis	1	Negative	----------	No	Stroke. Acute arthritis resolved.
12	74	V	no	Fever and low blood pressure	1	Negative	----------	No	Septic shock of unknown origin
13	29	M	She worked in cheese factory	Polyarthralgias	1	Negative	----------	Dx + Rf (3 months) + gentamicin (2 weeks)	Polyarthalgias
14	39	V	shepherd	Abdominal pain and fever	2	Negative	----------	Levofloxacin (8 days)	Self-limited unspecific abdominal pain.
15	50	V	No	Fever	1	Negative	Blood cultures (negatives)	Dx+ Rf (45 days)	Q fever
16	48	M	no	Fever	1	Negative	-----------	no	Self-limited febrile syndrome
17	58	V	no	Low back pain	1	Negative	PCR (negative), Blood cultures (negatives)	Dx (6 weeks) + gentamicin (14 days)	Prostate malignancy

Abbreviations: F: female; M: male; PCR: polymerase chain reaction; Dx: doxycycline; Stp: streptomycin; Rf: rifampin.

### Patients with a history of brucellosis

Only 5 out of the 17 patients had previously suffered from brucellosis. When the serology was negative for IgG and positive for IgM antibodies, the test was repeated for those patients between 2 and 11 times. The definitive diagnosis for each of these patients is shown in Table 1. Two patients in this group had suffered brucellosis over 20 years earlier. One of them was a 54 year old female veterinarian, but without direct contact with animals for at least the last 15 years. This patient had suffered acute brucellosis 20 years earlier with no relapses during the follow-up. The first determination of anti-*Brucella* antibodies by ELISA was requested for an occupational medical check-up while the patient was asymptomatic. The result showed IgM positive and IgG negative antibodies. ELISA testing was performed eight times for this patient. In two occasions sera were also positive for IgG and the other six tests were IgG negative and IgM positive. She did not receive antimicrobial therapy.

One other patient was an 82 year old woman. She had suffered brucellosis when she was 20 years old. Serology was requested due to a low back pain episode. Serology showed a pattern of positivity for IgM and negativity for IgG on two occasions. The final diagnosis was left renal carcinoma. She received no antibiotic treatment.

In the other 3 patients, the previous history of brucellosis was more recent. One was a 31 year old woman who had a diagnosis of brucellosis in 2005 because she presented fever as well as agglutinations, and she was PCR positive. During that year she received two cycles of treatment, first with doxycycline and rifampin, and later with doxycycline and streptomycin because of the reappearance of fever and arthralgia. In 2008, the patient received a new cycle of treatment with streptomycin and doxycycline because of the onset of musculoskeletal pain and she had a Wright sero-agglutination test titre value of 1/80. The patient was discharged in January 2009. But, a year and a half later, the patient consulted to the Rheumatology Department because of arthralgia. ELISA detection of antibrucella IgG and IgM antibodies was requested. From then until 2013, the ELISA testing continued being performed for this patient a total of 11 times, always obtaining the same IgG-negative, IgM-positive result.

Another patient was a 56 year old woman who was treated for brucellosis in 2006. She suffered from osteoarthritis and fibromyalgia and generalized joint pain. At that time, Wright agglutination was positive at titres of 1/40, and the Coombs anti-*Brucella* test was positive at titres 1/40. PCR detection for *Brucella* was positive. Later and during the follow-up, agglutinations, PCR detection and blood cultures were repeatedly negative until 2008. In 2011 an ELISA was requested, with the result of uncertain IgM and negative IgG on two occasions, and positive IgM and negative IgG on one occasion.

The last of the patients with a history of brucellosis was a 39-year-old man, who was treated in 2009 due to polyarthralgia and positivity in the Wright agglutination test with a titre of 1/160 and Coombs antibrucella with a titre 1/160. Thus, brucellosis was diagnosed despite the absence of epidemiological exposure history. After treatment with doxycycline and streptomycin, he was asymptomatic. In May 2010, another antibiotic treatment cycle was scheduled because of the appearance of arthralgia and fatigue. ELISA IgM was positive, but the patient did not improve with the treatment. Since 2009 ELISA was repeated 10 times during the follow-up always being IgM-positive and IgG-negative.

### Patients with no history of previous brucellosis

Of the 12 remaining patients, one was a shepherd, another patient worked in a dairy products factory, and the rest had no history of exposure to *Brucella* spp. Most of these patients had musculoskeletal symptoms as the cause that led to perform serology. Other reasons to perform serology were fever in four patients, and splenomegaly and pancytopenia in one patient. The Rose Bengal test was negative in all of these patients (table 2).

The number of samples with positive IgM and negative IgG was usually one. Four patients in this group received antibrucellar antibiotic treatment. One of them was treated with doxycycline for six weeks and also with streptomycin for the first two weeks. Another patient received doxycycline for six weeks and gentamycin for two weeks. One patient was treated with doxycycline, rifampin and gentamycin for the first two weeks and doxycycline and rifampicin until completing three months of treatment. Finally, one patient received doxycycline during 45 days as well as gentamycin during the first 14 days. The musculoskeletal symptoms of these patients did not improve with the use of antibiotics. Another two patients received antimicrobial therapy. One of these received doxycycline for only eight days and the other received levofloxacin.

The definitive clinical diagnosis of these patients is detailed in Table 2. Patients with fever were diagnosed as having a self-limited febrile syndrome in one case and septic shock of unknown origin in other case. In other two patients, the diagnosis of Q fever was established. Lymphoproliferative disorder was diagnosed in a patient with pancytopenia and splenomegaly as main clinical manifestations. A patient with low back pain and suspected spondylodiscitis was finally diagnosed as having vertebral metastases of a prostatic adenocarcinoma. Another patient was diagnosed of probable acute parvovirus infection. Unspecific self-limited abdominal pain was the diagnosis in another patient. The remaining four patients were diagnosed as having nonspecific degenerative or inflammatory musculoskeletal processes.

## Discussion

In our study we described a series of patients with suspected acute brucellosis in whom ELISA serology detected IgM but not IgG antibrucella antibodies. Most patients had symptoms related to the musculoskeletal system such as arthralgia or back pain. Seven of them were treated with antibiotics, but the clinical picture and the outcome were not suggestive of active infection caused by *Brucella* spp.

IgM antibodies are considered suggestive of acute infection and appear about a week after the onset of the disease, reaching a peak level one to three months later. IgG antibodies appear approximately three weeks after disease onset, reaching a maximum after six to eight weeks. Some studies give a specificity of 100% for the detection of IgM by ELISA for the diagnosis of acute brucellosis [17], [18]. However, other studies performed on the usefulness of different serological methods detected isolated cases with positive IgM in patients without brucellosis [11], [19]. In one of these studies the presence of cross-reactions was postulated, and the importance of a possible over-diagnosis in an area where other conditions such as malaria, tuberculosis, typhoid or rheumatoid arthritis can simulate clinical brucellosis was highlighted [11].

False positives in the determination of anti-*Brucella* IgM may be due to the presence of cross-reactions. These cross-reactions are due to antigenic similarity of the lipopolysaccharide of the cell wall with other Gram-negative bacteria. Cross-reactions with *Escherichia coli* O157, *Francisella tularensis*, *Yersinia enterocolitica*, *Vibrio cholerae* and *Salmonella* species have been described. Most of the antibodies responsible for these cross-reactions are IgM [19]. These cross-reactions are probably not responsible for the IgM antibrucella- antibodies in the patients of our series.

Furthermore, false positives in the determination of IgM antibodies may also be due to the presence of rheumatoid factor. Diaz *et al.*, described that situation in three cases of chronic hepatosplenic suppurative brucellosis. Although in two of these cases IgM antibrucella reactions were detected at first, the authors found that IgM became negative when the rheumatoid factor was pre-absorbed with an antiserum [15]. Although the frequency of rheumatoid factor in patients with brucellosis appears to be low, in those with chronic and focal disease that have a intense antigenic stimulation it may be more frequent. Mousa *et al.* described rheumatoid factor positivity in 8.8% of patients with osteoarticular brucellosis [20] and in only 0.2% of the patients without this complication. Although the above mentioned cases were patients with brucellosis, routine removal by pre-absorption of rheumatoid factor before determining *Brucella* IgM antibodies is recommended, as it may interfere with the test result [21]. The pre-absorption of rheumatoid factor was made in the sera samples from the patients of our series according to the instructions from the manufacturer of the commercial kit used.

Possible variability in the determination of antibodies between different commercial kits must be taken into account. Faadel *et al.* conducted a study in which they compared the results from four different commercial kits for the diagnosis of brucellosis by ELISA [22]. They used patient sera from Egypt and the United States. None of the commercial kits obtained a 100% specificity for neither IgM alone nor for IgM combined with IgG. Furthermore, slight differences in specificity were observed among patients from different locations, being slightly higher for sera from patients from Egypt. The authors conclude pointing out the importance of interpreting the results together with the patient's history, clinical features and other diagnostic test results.

False negatives can occur in cases of acute and early disease. One study found that up to 11% of patients with brucellosis had no detectable levels of specific IgM [23]. Negativity in some immunoassays may also be due to an excess of IgG antibodies [24]. It is therefore recommended the pre-absorption of these human sera to determine IgM antibodies [13]. Naha K *et al.* described a case of *Brucella suis* infection that was diagnosed using cultures from blood and bone marrow isolates, but serology was negative [25]. Possibly in this case, the use of extracts of lipopolysaccharide of *B. abortus* and *B. melitensis* in ELISA tests may give false negatives when the infection is caused by other *Brucella* species.

This is a retrospective study and therefore it is subject to some limitations. In most of the patients, it was not possible confirm the ELISA results by using other microbiological diagnostic methods. We also cannot rule out any false negative in determining IgG, mainly in those patients with previous brucellosis. However, diagnosis of active brucellosis in these patients may be ruled out with the medical history and clinical course data. Furthermore, our study reflects the real conditions in which clinical practice develops. We therefore believe that our results are valid and with clinical interest in the interpretation of *Brucella* serology by ELISA.

In conclusion, the detection of anti-*Brucella* IgM antibodies should not be regarded as definitive in the diagnosis of this infection. Prior to accepting this result as a true positive, the test should be repeated after pre-absorption of rheumatoid factor. Moreover, this finding should always be evaluated within the appropriate clinical history and epidemiological context and confirmed using another diagnostic method such as Brucella agglutination assay, as recommended by CDC [13], [26]. Our findings support the conclusion that, after detection of IgM anti-*Brucella* antibodies, the diagnosis of brucellosis must be confirmed by other methods.
